# Forget Me if You Can: Attentional capture by to-Be-remembered and to-Be-forgotten visual stimuli

**DOI:** 10.3758/s13423-016-1225-0

**Published:** 2017-01-10

**Authors:** Edyta Sasin, Candice C. Morey, Mark Nieuwenstein

**Affiliations:** 1 Department of Experimental Psychology, University of Groningen, Grote Kruisstraat 2/1, 9712 TS Groningen, The Netherlands; 2 Department of Psychology, University of Edinburgh, Scotland, UK

**Keywords:** Directed forgetting, Attentional capture, Working memory

## Abstract

Previous studies on directed forgetting in visual working memory (VWM) have shown that, if people are cued to remember only a subset of the items currently held in VWM, they will completely forget the uncued, no longer relevant items. While this finding is indicative of selective remembering, it remains unclear whether directed forgetting can also occur in the absence of any concurrent to-be-remembered information. In the current study, we addressed this matter by asking participants to memorize a single object that could be followed by a cue to forget or remember this object. Following the cue, we assessed the object’s activation in VWM by determining whether a matching distractor would capture attention in a visual search task. The results showed that, compared to a cue to remember, a cue to forget led to a reduced likelihood of attentional capture by a matching distractor. In addition, we found that capture effects by to-be-remembered and to-be-forgotten distractors remained stable as the interval between the onset of the cue and the search task increased from 700 ms to 3900 ms. We conclude that, in the absence of any to-be-remembered objects, an instruction to forget an object held in WM leads to a rapid but incomplete deactivation of the representation of that object, thus allowing it to continue to produce a weak biasing effect on attentional selection for several seconds after the instruction to forget.

*“Forgetting is as important a function as remembering.”* (p. 148; James, 1892).

As alluded to by William James, there are benefits to forgetting. In the domain of working memory (WM), the mind’s system for temporary maintenance and manipulation of information (Baddeley, 2010; Carlisle, Arita, Pardo, & Woodman, 2011; Cowan, 2008; Oberauer et al. 2012), forgetting is important because WM is known to be limited in its capacity for retaining information (Cowan, 2010; Luck & Vogel, 1997) and because it is known that information activated in WM biases the selection of new perceptual input towards matching stimuli (Desimone & Duncan, 1995; Downing, 2000; Greene, Kennedy, & Soto, 2015; Olivers, Meijer, & Theeuwes, 2006; Pan, 2010; Sasin, Nieuwenstein, & Johnson, 2015; Sasin & Nieuwenstein, 2016; Soto, Heinke, Humphreys, & Blanco, 2005). Thus, the forgetting of no longer relevant information is of importance to protect WM from being overloaded and to prevent no longer relevant information from guiding attentional selection and behavior (Ecker, Lewandowsky, & Oberauer, 2014; Ecker, Oberauer, & Lewandowsky, 2014).

In previous studies, directed or intentional forgetting of information in WM has been investigated using the retro-cuing paradigm (Gunseli, Van Moorselaar, Meeter, & Olivers, 2015; Olivers, Meijer, & Theeuwes, 2006; Pertzov, Bays, Joseph, & Husain, 2013; Van Moorselaar, Battisoni, Theeuwes, & Olivers, 2015; Van Moorselaar, Olivers, Theeuwes, Lamme, & Sligte, 2015; Williams, Hong, Kang, Carlisle, & Woodman, 2013; Williams & Woodman, 2012). In this paradigm, participants are first presented an array of items that have to be encoded into WM and then they are presented with a cue that indicates which subset of these items needs to be prioritized and remembered for a later memory test. Subsequently, on some trials, memory for the uncued items is unexpectedly assessed, either by asking participants to reproduce an uncued item or by examining whether a distractor that matches one of the uncued items captures attention in a visual search task, a phenomenon known as memory-driven attentional capture (Downing, 2000; Greene, Kennedy, & Soto, 2015; Olivers, Meijer, & Theeuwes, 2006; Pan, 2010; Sasin, Nieuwenstein, & Johnson, 2015; Soto, Heinke, Humphreys, & Blanco, 2005). Taken together, the results of these studies show that retro-cues are effective in enhancing memory for the cued items, and they make clear that this enhancement goes at the expense of memory for the uncued items, as many of these studies found that the uncued items are completely forgotten. Specifically, studies employing the implicit measure of memory-driven attentional capture show no evidence of capture by distractors matching an uncued item (Olivers et al., 2006; van Moorselaar, Battistoni, et al. 2015; van Moorselaar, Olivers, et al. 2015), whereas studies using explicit measures show that participants can only guess in trying to reproduce the color of an uncued item (Williams et al., 2013). In accounting for these effects, it has been proposed that a retro-cue leads to attentional prioritization and selective maintenance of the cued items. That is, in more general terms, the cue is assumed to cause of shift or redistribution of limited WM resources to only the cued items (see, also, Bays & Husain, 2008), thus causing the uncued items to decay in the absence of resources to support their retention in WM.

## The current study

While studies using the retro-cuing paradigm provide compelling evidence that people are capable of selectively remembering a cued subset of items held in visual working memory (VWM), they leave unresolved whether people are able to intentionally forget no-longer relevant items held in VWM. That is, the fact that people can selectively remember a cued subset of items does not entail intentional forgetting of the uncued items, as the forgetting of uncued items could be explained as an unintentional consequence of the attentional prioritization of the cued items. In the current study, we set out to provide a more direct test of intentional forgetting by examining how a cue to forget an earlier memorized object influences attentional capture by a matching distractor when there is no competing information to be retained in VWM. Specifically, we asked participants to memorize a single colored shape which was followed by a cue that indicated whether this object had to be retained in VWM for a later recognition test or whether it could be forgotten. To ensure that participants would adhere to the instruction provided by the retro-cue, the cue was 100% valid (Gunseli et al., 2015, Williams & Woodman, 2012), meaning that we never unexpectedly probed participants for their memory of the uncued item in a surprise recognition test. To determine whether a cue to forget would indeed lead to intentional forgetting, we used an implicit measure of VWM activation by examining whether a distractor that matched the to-be-remembered or to-be-forgotten object would capture attention in an unrelated visual search task that was performed at different intervals following the cue. By varying the duration of the interval separating the cue and the visual search task, we aimed to assess the time course of any cue-induced forgetting.

## Experiment 1

### Method

#### Participants

Eighteen students of the University of Groningen (11 females; M = 20.3 years; SD = 1.45) participated in the experiment for partial course credit. All participants had normal or corrected to normal visual acuity. The study was approved by the Ethics Committee of the Psychology Department. Informed written consent was obtained.

#### Apparatus and stimuli

Stimulus presentation and response collection were controlled by a program written with E-prime 2.0 (Schneider, Eschmann, & Zuccolotto, 2002) and the experiment was conducted on computers that were fitted with 22-inch (c.56-cm) CRT computer monitors with a refresh rate of 100 Hz and a resolution of 1024 × 768 pixels.

All stimuli were presented on a gray background. The shapes used were a circle (1.8° × 1.8° of visual angle), a diamond (1.8° × 1.8°), a square (1.9° × 1.9°), a triangle (2° × 1.7°), and a hexagon (2° × 1.7°). The color of the shapes could be red (R = 255, G = 0, B = 0), green (R = 0, G = 255, B = 0), blue (R = 0, G = 0, B = 255), yellow (R = 255, G = 255, B = 0) or pink (R = 255, G = 192, B = 203). The thickness of the border line of the shapes was 0.12° in visual angle. One colored shape was displayed as a memory object at the center of the screen. Four colored shapes were displayed for the search task and each shape contained a black line (0.57° length × 0.12°). The three distractor lines were vertical and the target line was tilted 38° either to the left or to the right. The shapes were positioned at the corner of an imaginary rectangle measuring 5.7° of visual angle horizontally and 4.1° vertically. Each object in the search display was unique in color and shape. The cue to forget or remember was displayed in the form of the corresponding word being shown in black Courier New, 20-point font, at the center of the screen.

#### Procedure

**Fig. 1:**
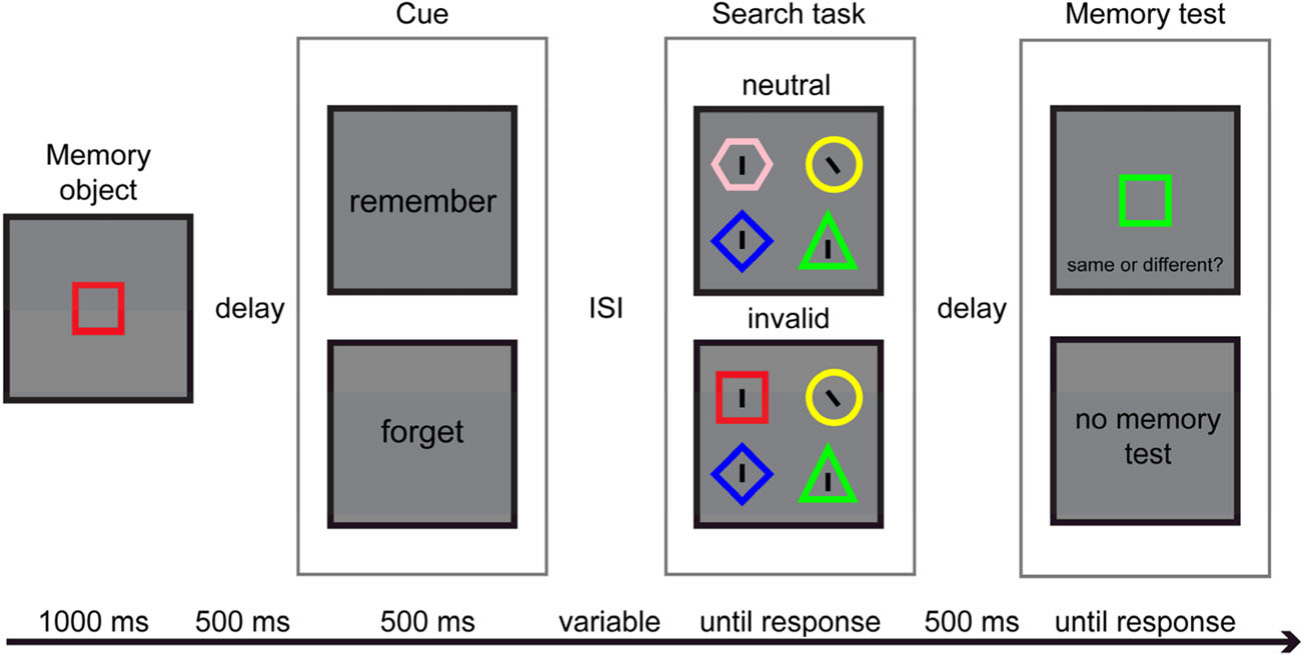
Illustration of the task used in Experiment 1 and Experiment 2. Presentation of memory object was followed by the word cue (*forget* or *remember*). After variable ISI (*200 ms*, *600 ms*, *1000 ms* or *1400 ms* in Experiment 1 and *200 ms*, *600 ms*, *2000 ms* or *3400 ms* in Experiment 2, participants performed the search task. The memory task was present only on trials with the cue *remember*

Figure 1 illustrates different types of trials used in Experiment 1. At the beginning of each trial, there was 500-ms fixation period, after which the memory object was presented for a duration of 1000 ms. Participants were asked to remember both the color and the shape of the memory object. Next, there was a 500-ms blank interval followed by the cue which indicated whether the memory object had to be maintained in memory for a later memory test or whether it could be forgotten. The cue was displayed for 500 ms. After an inter-stimulus-interval (ISI) of 200, 600, 1000 or 1400 ms, the search display appeared. The participant’s task for the search display was to discriminate the orientation of the target line by pressing the “Z” key when it was tilted to the left and by pressing the “M” key when it was tilted to the right. There were two different types of trials. On *invalid* trials, one of the objects in the search display matched both the color and the shape of the memory object. This matching object always contained a distractor line and always was presented on the opposite side to the target. On *neutral* trials, neither of the features of the memory object was shared by any of the objects in the search display. The search display remained on the screen until the response was made. The response was followed by 500-ms blank interval. In the remember condition, the memory test followed the offset of this blank interval. For this test, a colored shape appeared in the center of the screen and participants were instructed to indicate whether this memory probe was identical (in shape and color) to the memory object. On *different* trials, the memory probe and the memory object differed in color, shape or both features and the participants had to respond by pressing the “M” key of the keyboard. When the memory probe was the same as the memorized object, participants had to press the “Z” key. Participants were instructed to execute the memory task as accurately as possible, without time pressure. The search task had to be completed as quickly and accurately as possible.

The manipulations of cue (*remember* or *forget*), distractor match (*invalid* or *neutral*) and ISI (*200, 600, 1000* or *1400* ms) resulted in a total of 16 conditions which were presented in a random order. There were 32 trials for each condition, resulting in a total of 512 trials which were presented in blocks of 64 trials each. The experiment was preceded by 32 trials to practice the tasks and the experiment took about 55 min in total.

## Results

Performance was near ceiling in the search and memory tasks (M = 98% and M = 94% correct, respectively). Before analyzing the RTs for the search task, we first excluded those trials from the remember condition in which the response to the memory task was incorrect. In addition, we excluded all trials in which the response to the search task was incorrect, and we also excluded trials in which the search-RT appeared to be an outlier. Specifically, we first excluded trials with search-RTs shorter than 150 ms or longer than 3000 ms, and we subsequently identified and excluded any remaining outliers using the procedure described by Van Selst and Jolicoeur (1994). Together, these procedures resulted in a loss of 2.4% of data points. The exclusion of trials did not change the pattern of results.

**Fig. 2:**
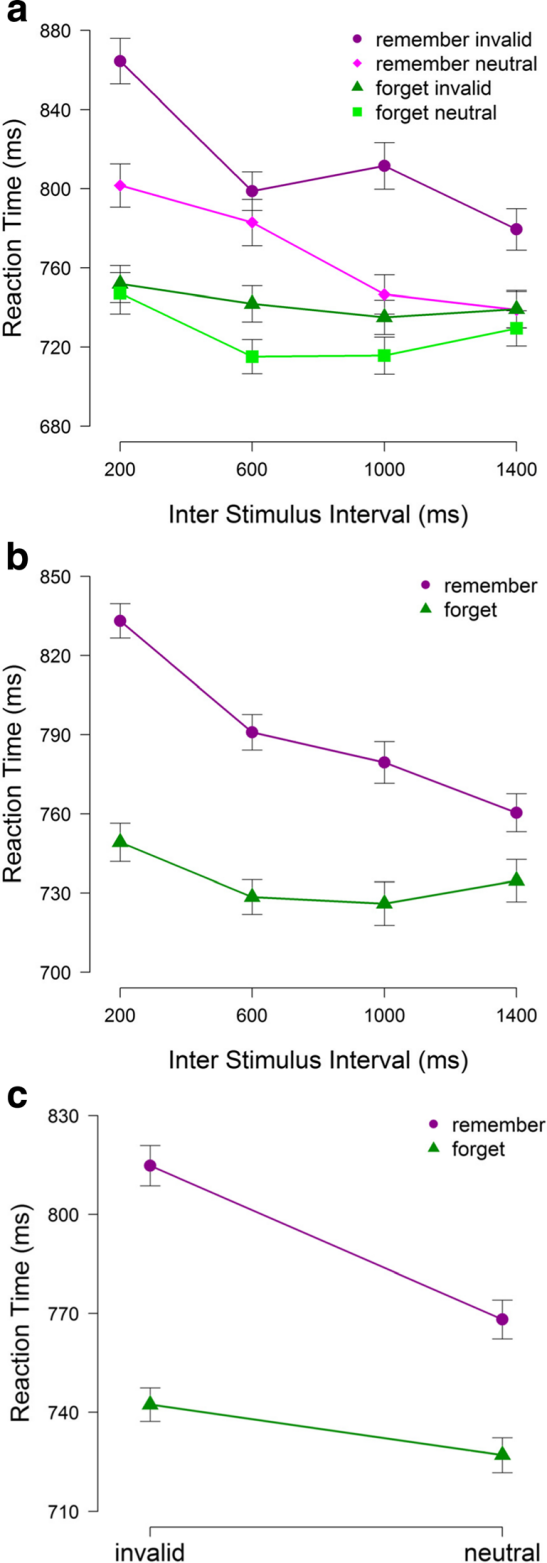
Results of Experiment 1. Mean RTs (ms) in the search task as a function of cue condition, match condition and the ISI (*A*). Mean RTs (ms) in the search task as a function of cue condition and ISI (*B*). Mean RTs (ms) in the search task as a function of cue condition and match condition (*C*). *Error bars* within-subject standard errors of the mean (Morey, 2008)

To examine performance on the search task, we conducted a 2 (cue: remember or forget) × 2 (match: invalid or neutral) × 4 (ISI: 200, 600, 1000 or 1400 ms) repeated measures ANOVA on the mean RTs for correct responses. The results, illustrated in Fig. 2, revealed a main effect of cue, *F*(1,17) = 22.33, *p* < .001, *η*_*p*_^2^=.57, match, *F*(1,17) = 27.01, *p* < .001, *η*_*p*_^2^=.61, and ISI, *F*(3,51) = 7.24, *p* = .003, *η*_*p*_^2^=.30. In addition, there were significant interactions of cue and ISI, *F*(3,51) = 2.91, *p* = .049, *η*_*p*_^2^=.15, and of cue and match, *F*(1,17) = 10.87, *p* = .004, *η*_*p*_^2^=.39. The two-way interaction between match and with ISI was non-significant, *F*(3,51) = 1.09, *p* = .355, *η*_*p*_^2^=.06, and the three-way interaction between match, cue and ISI was also not significant, *F*(3,51) = 1.16, *p* = .325, *η*_*p*_^2^=.06.

**Fig. 3:**
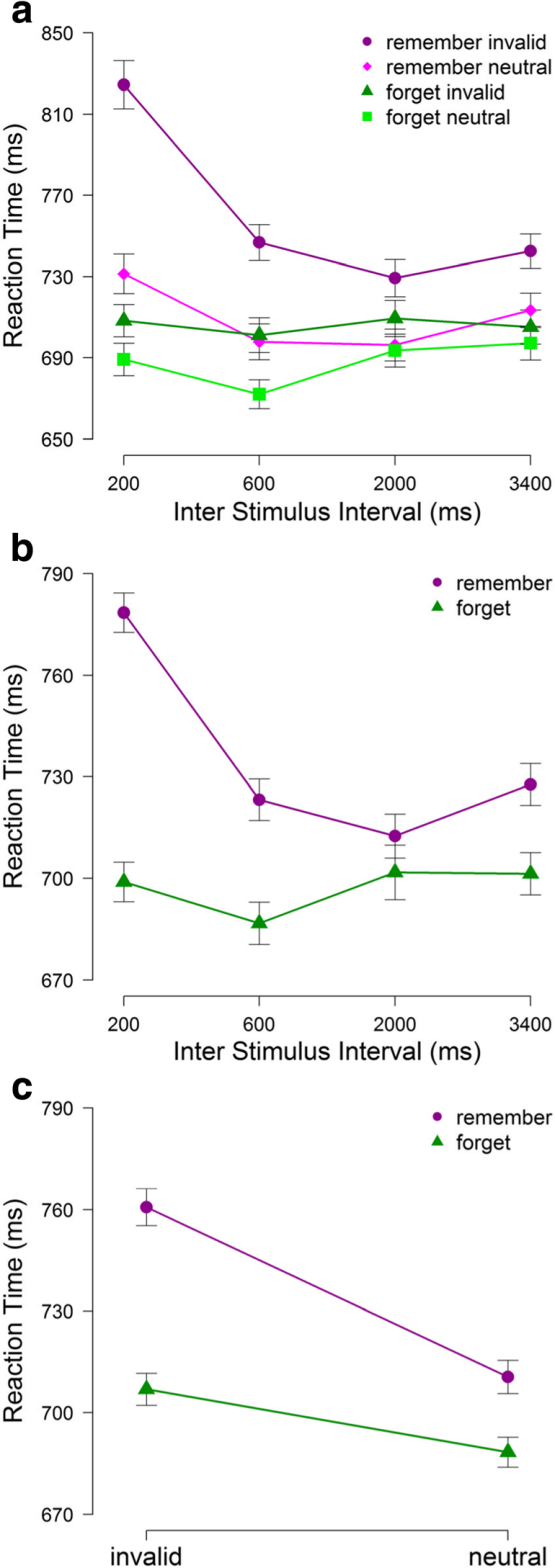
Results of Experiment 2. Mean RTs (ms) in the search task as a function of cue condition, match condition and the ISI (*A*). Mean RTs (ms) in the search task as a function of cue condition and ISI (*B*). Mean RTs (ms) in the search task as a function of cue condition and match condition (*C*). *Error bars* within-subject standard errors of the mean (Morey, 2008)

In a subsequent series of analyses, we further examined the two-way interactions between cue and ISI, and between cue and match. In examining the interaction between cue and ISI (see Fig. 3), we first tested the effect of ISI for each cue condition separately. These analyses showed that there was a significant effect of ISI in the remember condition *F*(3,51) = 7.24, *p* = .001, *η*_*p*_^2^=.30, but not in the forget condition, *F*(3,51) = 1.30, *p* = .284, *η*_*p*_^2^=.07. For the remember condition, a series of post hoc comparisons using the Holm–Bonferroni correction revealed that RTs were significantly slower at the ISI of 200 ms than at all longer ISIs (all *t*’s > 2.11, all *p*’s < .047), with no significant differences in RT for ISIs of 600 ms and longer (all *t*’s < 1.89, all *p*’s > .485). A similar follow-up analysis for the interaction between cue and match showed that invalid trials resulted in slower RTs relative to neutral trials for both the remember condition, M = 826 versus M = 774 ms, respectively, *t*(17) = 6.01, *p* < .001, and the forget condition, 758 ms versus 741 ms, respectively, *t*(17) = 2.94, *p* = .009, with the magnitude of the capture effect being significantly larger in the former (52 ms) than in the latter condition (16 ms), *t*(17) = 4.10, *p* = .001.

## Experiment 2

Taken together, the results of Experiment 1 show that a distractor matching an earlier memorized object captured attention regardless of whether it had to be remembered or forgotten, but the capture effect was smaller when the object had to be forgotten. Furthermore, the results of Experiment 1 showed that the likelihood of attentional capture by a distractor matching the to-be-forgotten object remained constant across ISIs of 200– 1400 ms, thus suggesting that there was no decay of the to-be-forgotten object across this time period. To test if any such decay would occur with longer intervals, Experiment 2 replicated Experiment 1 with the exception that the ISI between the cue and search task was 200, 600, 2000 or 3400 ms.

## Method

### Participants

Eighteen of the University of Groningen (12 females; M = 20.6 years; SD = 2.19) participated in the experiment for partial course credit. None of the participants had taken part in Experiment 1 and all participants had normal or corrected to normal visual acuity. The study was approved by the Ethics Committee of the Psychology Department. Informed written consent was obtained.

### Apparatus and stimuli

The stimuli and apparatus were identical to those used in Experiment 1.

### Procedure

The procedure was identical to Experiment 1 except that the ISI between the word cue and the search task was 200, 600, 2000 or 3400 ms.

## Results

Participants achieved 92% correct on the memory task and 97% correct on the search task. In analyzing the results from the search task, we followed the same procedures as those described for Experiment 1, resulting in a loss of 2.5% of the data points. The exclusion of trials did not change the pattern of results.

Performance on the search task was analyzed in the same way as in Experiment 1. The results, illustrated in Figure 3 showed significant main effects of cue, *F*(1,17) = 27.60, *p* < .001, *η*_*p*_^2^=.62, match, *F*(1,17) = 26.94, *p* < .001, *μ*_*p*_^2^=.61, and ISI, *F*(3,51) = 6.83, *p* = .002, *η*_*p*_^2^=.29. In addition, the results revealed significant interactions between cue and ISI, *F*(3,51) = 7.72, *p* = .002, *η*_*p*_^2^=.31, and between cue and match, *F*(1,17) = 11.90, *p* = .004, *η*_*p*_^2^ =.41. There was no interaction between match and ISI, *F*(3,51) = 2.67, *p* = .071, *η*_*p*_^2^=.14, nor between match, cue, and ISI, *F*(3,51) = 2.57, *p* = .084, *η*_*p*_^2^ = 13.

In a follow-up analysis, we further examined the Cue × ISI interaction by testing the effect of ISI for the remember and forget conditions separately. The effect of ISI was significant in the remember condition, *F*(3,51) = 13.71, *p* < .001, *η*_*p*_^2^ =.45, but not in the forget condition, *F*(3,51) = 0.87, *p* = .464, *η*_*p*_^2^ =.05. For the remember condition, post hoc comparisons with the Holm–Bonferroni correction revealed that RTs were significantly slower at the ISI of 200 ms than at all longer ISIs, all *t*’s > 2.11, all *p*’s < .047, with no significant differences in RT across ISIs of 600-3400 ms, all *t*’s < 0.95, all *p*’s > .542. To further examine the Cue × Match interaction (see Fig. 2), we conducted paired-samples *t* tests. For the remember condition, RTs were slower in the invalid (761 ms) than in the neutral (711 ms) condition, *t*(17) = 5.98, *p* < .001, and the same was true for the forget condition (707 ms vs. 688 ms, respectively; *t*(17) = 2.36, *p* = .030). The difference in RT on invalid and neutral trials was significantly smaller in the forget condition (19 ms) than in the remember condition (50 ms), *t*(17) = 3.35, *p* = .004.

## General discussion

Previous studies on directed forgetting in VWM have shown that, if participants are cued to remember a subset of items currently held in VWM, their memory for the cued subset will be enhanced at the expense of forgetting the uncued items (Olivers et al., 2006; Van Moorselaar et al., 2015a; Van Moorselaar et al., 2015b; Williams et al., 2013; Williams & Woodman, 2012). While this finding makes clear that people can selectively remember a cued subset of information held in VWM, it leaves unresolved whether people can also intentionally forget no longer relevant information in the absence of any concurrent to-be-remembered information. To address this matter, we asked participants to memorize a single object which was followed by a cue to either remember or forget this object, and we examined the effects of this instruction to remember or forget by determining the likelihood that a distractor matching the earlier memorized object would capture attention in a visual search task that was presented at different intervals following the cue. The results of two such experiments converged in showing that an instruction to forget leads to deactivation of the memory trace of the earlier memorized object, such that attentional capture was still present but less pronounced following an instruction to forget, as opposed to remember, the earlier memorized object. Furthermore, the results showed that capture was unaffected by the duration of the interval between the cue and the search task, and they showed that an instruction to remember led to slower visual search when the search task followed closely in time to the instruction to remember whereas no such slowing of search was observed following a cue to forget.

In demonstrating that, in the absence of any to-be-remembered items, a cue to forget an earlier memorized object leads to a reduced likelihood of memory-driven attentional capture, the current findings move beyond the results of previous studies which showed evidence of complete forgetting of to-be-forgotten objects in the presence of to-be-remembered objects (Olivers et al., 2006; Van Moorselaar et al., 2015a; Van Moorselaar et al., 2015b; Williams et al., 2013; Williams & Woodman, 2012). Specifically, while the forgetting of uncued materials in previous studies can be explained as a side effect of the attentional prioritization and selective maintenance of to-be-remembered items, the current finding that an instruction to forget an earlier memorized object led to a reduced likelihood of attentional capture by a matching distractor can only be explained in terms of intentional forgetting.

In accounting for why intentional forgetting did not fully prevent the occurrence of memory-driven attentional capture, we can conceive of three possible explanations: Participants might have occasionally failed to adhere to the instruction to forget, the to-be-forgotten object might have guided attention based on a long-term memory (LTM) representation, or forgetting might have led to an incomplete deactivation of the WM trace, thus allowing for a weak, residual guidance effect. In considering these three possibilities in closer detail, it becomes clear that incomplete deactivation appears to provide the least contentious explanation. Specifically, the notion that participants sometimes failed to adhere to the cue to forget seems incompatible with a post-hoc analysis of the variance of RTs in the forget condition. In this analysis, we compared the standard deviations of RTs in the invalid and neutral trials of the forget condition, the idea being that variance should be larger on invalid trials than neutral trials if the invalid trials indeed included some trials in which capture occurred because of unsuccessful forgetting. The results of this comparison, however, revealed no difference in the standard deviations of RTs,^1^ thus providing no evidence for the possibility that the residual attentional capture effect seen in the forget condition stemmed from an occasional failure to forget the earlier memorized object. With regard to the possibility that this residual capture effect was driven by a long-term memory representation, we note that while the repeated exposure and memorization of stimuli might indeed have led to an LTM representation which could potentially guide visual attention (Rosen, Stern, Michalka, Devaney, & Somers, 2015; Woodman, Carlisle, & Reinhart, 2013), this seems an unlikely account of the residual capture effect because any such LTM-guidance would be expected to occur for all distractors in the search task, thus precluding an LTM-driven capture effect as an explanation for the capture effect found only in invalid trials in the forget condition. By implication, it seems that the residual capture effect found in the forget condition is best explained in terms of an intentional but incomplete deactivation of the representation of the to-be-forgotten object. In this regard, the current findings can be said to corroborate the results of studies of intentional forgetting of verbal information, which have also provided evidence that people can choose to forget verbal information from WM by demonstrating that increasing the number of forget cues leads to more complete forgetting (Anderson & Green 2001; see also, Lee, Lee, & Tsai, 2007). Indeed, in light of these earlier findings, it can be argued that the current finding that memory-driven attentional capture still occurred several seconds after a cue to forget may be due to the fact that our use of a single cue to forget was insufficient to cause complete forgetting of the earlier memorized object.

In considering the mechanism underlying the current finding of directed forgetting, it is of relevance to note that, while the capture effect by distractors matching the to-be-forgotten object remained stable as the interval between the cue and the search task increased from 200 to 3400 ms, we also did not find evidence for a cost of processing the cue to forget, such that reaction times on the search task were found to be stable as the interval between the cue and the search task increased from 200 to 3400 ms. In other words, the current findings show that the processing of the cue to forget did not incur a cost to performance on the search task, and they show that the residual activation of the to-be-forgotten object did not dissipate across increasing delays. In considering the implications of these findings, it is important to bear in mind that the cue itself was presented for 500 ms, meaning that the shortest interval between the onset of the cue and the onset of the search task was 700 ms. Thus, it appears to be the case that the partial forgetting that was triggered by the cue took effect during this 700-ms interval, such that no further forgetting occurred across longer intervals. In this regard, the current findings can be said to argue against decay-based theories of forgetting (e.g., Barrouillet, Bernardin, & Camos, 2004), whereas they offer support for interference-based theories of forgetting (e.g., Oberauer, Lewandowsky, Farrell, Jarrold, & Greaves, 2012), as the former would predict that forgetting should increase with additional time, whereas the latter would not predict such an effect if that period of time is not used for processing other information, as was the case in the current study.

In the current study, we found that an instruction to forget the earlier encoded object led to overall faster performance in a subsequent, unrelated search task that was performed at different intervals after the presentation of the cue to forget the object. This finding differs from the finding reported by Fawcett and Taylor (Fawcett & Taylor, 2008; 2010), who found that an instruction to forget an earlier encoded word resulted in slower responses for a subsequent unrelated task when the interval separating the cue and this unrelated task was less than 1800 ms. In accounting for the discrepancy between the current finding and this finding by Fawcett and Taylor, it is important to note that there are a number of methodological differences that might explain this discrepancy. Specifically, the study by Fawcett and Taylor presented participants with a study word on each trial, followed by an instruction to remember or forget. Subsequently, participants performed unrelated detection tasks. After all the study words were presented, participants performed a recognition test examining memory for the to-be-remembered words and, unexpectedly, for the to-be-forgotten words as well. In contrast, we asked participants to memorize a simple colored shape on each trial, which was followed by an instruction to remember or forget which in turn was followed by the unrelated search task. After the search task, a memory test was presented only on trials in which participants were cued to remember the object. In considering which of these methodological differences might explain the discrepancy in results, one could argue that perhaps the use of verbal materials and the requirement to forget in the presence of to-be-remembered words might have led to different results in the study by Fawcett and Taylor. Specifically, it could be that participants in the study by Fawcett and Taylor required more time to process the instruction to forget due to the difficulty of forgetting a word, as opposed to forgetting one of a small set of possible colored shapes, and due to the extra time and effort it might take to forget in the presence of to-be-remembered items. Indeed, it is worth noting that Ecker and colleagues (Ecker, Lewandowsky, et al. 2014; Ecker, Oberauer, et al. 2014) found that it takes less time to remove three items from WM than it takes to remove only one or two of these items, thus lending credence to the possibility that the discrepancy between the current study and the findings by Fawcett and Taylor could be due to the additional time it takes to forget in the presence of to-be-remembered items.

To conclude, the current study shows that memory-driven capture offers a highly useful paradigm to study intentional forgetting of information held in VWM. Supplementing previous findings, the current findings show that participants can choose to forget a single object held in VWM even when there are no concurrent processing demands. In addition, they show that such forgetting need not result in a complete deactivation of the object’s representation in WM, and they suggest that the process by which forgetting occurs can be accomplished rapidly, within 700 ms after the appearance of an instruction to forget an earlier memorized object.

## Footnotes

1 Specifically, we found that the average SD of RTs was equal for neutral and invalid trials in the forget condition, M = 173 vs. M = 183, *t*(35) = 1.80, *p* = .081.
